# The natural history study of preclinical genetic Creutzfeldt-Jakob Disease (CJD): a prospective longitudinal study protocol

**DOI:** 10.1186/s12883-023-03193-8

**Published:** 2023-04-14

**Authors:** Noa Bregman, Tamara Shiner, Gitit Kavé, Roy Alcalay, Mali Gana-Weisz, Orly Goldstein, Tal Glinka, Orna Aizenstein, Dafna Ben Bashat, Yifat Alcalay, Anat Mirelman, Avner Thaler, Nir Giladi, Nurit Omer

**Affiliations:** 1 Cognitive Neurology Unit, Neurological Institute, Tel-Aviv Medical Center, Tel-Aviv, Israel; 2 Sackler School of Medicine, Tel-Aviv University, Tel-Aviv, Israel; 3 Sagol School of Neuroscience, Tel-Aviv University, Tel-Aviv, Israel; 4 Department of Education and Psychology, The Open University, Ra’anana, Israel; 5 Laboratory of biomarkers and genomic of neurodegeneration, Tel-Aviv Medical Center, Tel-Aviv, Israel; 6 Sagol Brain Institute, Wohl Institute for Advanced Imaging, Sourasky Medical Center, Tel Aviv, Israel; 7 Department of Diagnostic Imaging, Sourasky Medical Center, Tel Aviv, Israel; 8 Division of Clinical Laboratories, Tel Aviv Sourasky Medical Center, Tel-Aviv, Israel; 9 Laboratory of early markers of neurodegeneration, Neurological Institute, Tel-Aviv Sourasky Medical Center, Tel-Aviv, Israel; 10 Brain Institute, Tel Aviv Sourasky Medical Center, Tel Aviv, Israel

**Keywords:** Creutzfeldt-Jakob Disease, Genetic Creutzfeldt-Jakob Disease, Prion disease, Prodromal CJD, Biomarkers, E200K, Neurodegeneration, Protocol, Phenoconversion

## Abstract

**Background:**

Creutzfeldt-Jakob Disease (CJD) is the most common prion disease in humans causing a rapidly progressive neurological decline and dementia and is invariably fatal. The familial forms (genetic CJD, gCJD) are caused by mutations in the *PRNP* gene encoding for the prion protein (PrP). In Israel, there is a large cluster of gCJD cases, carriers of an E200K mutation in the *PRNP* gene, and therefore the largest population of at-risk individuals in the world. The mutation is not necessarily sufficient for the formation and accumulation of the pathological prion protein (PrP^sc^), suggesting that other, genetic and non-genetic factors affect the age at symptoms onset. Here we present the protocol of a cross-sectional and longitudinal natural history study of gCJD patients and first-degree relatives of gCJD patients, aiming to identify biological markers of preclinical CJD and risk factors for phenoconversion.

**Methods:**

The study has two groups: Patients diagnosed with gCJD, and first-degree healthy relatives (HR) (both carriers and non-carriers of the E200K mutation in the *PRNP* gene) of patients diagnosed with gCJD. At baseline, and at the end of every year, healthy participants are invited for an “in-depth” visit, which includes a clinical evaluation, blood and urine collection, gait assessment, brain MRI, lumbar puncture (LP), and Polysomnography (PSG). At 6 months from baseline, and then halfway through each year, participants are invited for a “brief” visit, which includes a clinical evaluation, short cognitive assessment, and blood and urine collection. gCJD patients will be invited for one “in-depth” visit, similar to the baseline visit of healthy relatives.

**Discussion:**

This continuous follow-up of the participants and the frequent assessments will allow early identification and diagnosis in case of conversion into disease. The knowledge generated from this study is likely to advance the understanding of the underlying clinicopathological processes that occur at the very beginning of CJD, as well as potential genetic and environmental risk factors for the development of the disease, therefore advancing the development of safe and efficient interventions.

**Trial registration:**

The study is an observational study. It has registered retrospectively in https://clinicaltrials.gov/ and has been assigned an identification number NCT05746715.

**Supplementary Information:**

The online version contains supplementary material available at 10.1186/s12883-023-03193-8.

## Background

Creutzfeldt-Jakob Disease (CJD) is the most common prion disease in humans. It results from a conformational change of the normal prion protein (PrP^c^) into a pathological protein (PrP^sc^) that aggregates in the central nervous system [1]. Once formed, the initial PrP^sc^ oligomers can act as seeds for further misfolding of normal cellular PrP^c^ in a cascade that leads to neuronal death, as well as to the clinical manifestations of the disease.

Early symptoms are nonspecific and may include headaches, anxiety, and behavioral changes. Later, diffuse neurological impairment appears, including cognitive decline, cerebellar ataxia, pyramidal, and extrapyramidal deficits, disturbances of vision, and myoclonus. CJD can be etiologically classified into transmitted, sporadic (sCJD), and genetic forms (gCJD). The familial forms are caused by mutations in the PRNP gene that encodes for the prion protein (PrP). The largest cluster of gCJD cases exists in Jews of Libyan and Tunisian ancestry, carrying an E200K mutation (Glu to Lys substitution) in the *PRNP* gene. Approximately 200,000 Libyan Jews live in Israel, resulting in the largest population of at-risk individuals in the world [2].

The disease is invariably fatal, and survival is between several months to 1 year [3], with a mean survival from onset to death of 7.6 months [4]. Thus, by the time the disease is diagnosed, the neural pathology is extensive, and the neurodegeneration is vast. The presence of a genetic mutation may help identify individuals at risk for developing the disease as the penetrance of the mutation approaches 100% [5]. Carriers of the E200K mutation are born with this dominantly inherited mutation, but most remain asymptomatic until middle age, and some develop the disease as late as 80 years old [5].

E200K-PrP is initially formed as a normal PrP^c^ isoform [6]. Therefore, the mutation is not necessarily sufficient for the formation and accumulation of PrP^sc (6)^. This fact suggests that other, genetic and non-genetic factors affect the age at symptoms onset. The *CYP4 × 1* gene may influence the age at onset in patients with gCJD due to the E200K mutation and in sCJD [7]. Inheritance of gCJD with E200K mutation may exhibit anticipation (an earlier symptom onset or more severe presentation in offspring) [8]. However, this claim requires further investigation [9]. Other modifiers remain obscure.

Definitive diagnosis of CJD requires autopsy or brain biopsy. Clinical diagnostic criteria for CJD include a combination of cerebrospinal fluid (CSF) biomarkers, magnetic resonance imaging (MRI), electroencephalography (EEG), and clinical symptoms [10]. Early diagnosis of CJD remains challenging because the clinical manifestations of prion diseases at onset are variable and non-specific. Recently, testing of CSF with new in-vitro PrP^sc^ amplification technology, and designated real-time quaking-induced conversion (RT-QuIC), has shown considerable promise as a highly specific and sensitive tool for the diagnosis of sCJD [11]. RT-QuIC is a protein aggregation assay using quaking to induce misfolding of normal prions into PrP^sc^, with simultaneous fluorescent readout [12]. CSF RT-QuIC has proven to be a relatively sensitive (73-97%) [13, 14] and a highly specific biomarker (99-100%) [12, 15–17] for diagnosing CJD, and it is now part of the standard diagnostic criteria [10].

As with many neurodegenerative diseases, subtle clinical symptoms are likely to present even before diagnostic criteria are met. Based on the manifestation at the time of diagnosis, several clinical symptoms and signs seem to be common in most patients. These include visual deficits, balance and gait changes, sleep disturbances, as well as cognitive and behavioral impairments. Thus, it is possible that a more sensitive assessment could detect these changes before diagnosis.

Presently, diagnosis at the pre-clinical or prodromal stage is not feasible, as no validated blood biomarker for the prediction of symptoms onset exists. Previous attempts have been made to characterize the prodromal stage. Lee et al. tested a relatively large number of E200K asymptomatic carriers for a longitudinal neuroimaging study, and described clinical and radiological data of pheno-convertors. Their results show that MRI imaging by itself may not be sufficiently sensitive to detect changes during the pre-symptomatic stage [18]. In the attempt to find surrogate markers in the serum and CSF of patients with CJD or those that are at risk of developing the disease, Steinacker et al. reported increased neurofilaments (NF’s) and Tau protein in the serum of patients with CJD. In one asymptomatic carrier of prion disease, NF’s in CSF were elevated before symptoms’ onset [19], raising the question of their role as early biomarkers that may predict disease onset.

It has become increasingly evident that sleep dysfunction commonly accompanies chronic neurodegenerative conditions and may predate the onset of overt symptoms of these disorders by several years [20–23]. For example, rapid eye movement (REM) sleep behavior disorder (RBD) may precede the development of a synucleinopathy by decades [24–27], and reduced sleep efficiency is seen in patients with preclinical AD [28]. Sleep disturbances have not been included as a principal symptom of CJD [29]. However, prior studies on sleep characteristics in CJD demonstrate that sleep disturbances are common in patients with CJD [30–32], with one study showing nearly 90% of patients with at least one sleep-related complaint at the time of their initial evaluation. The most frequently observed polysomnography (PSG) aberrations were loss of normal sleep EEG architecture and the presence of sleep-disordered breathing. 38% of patients who achieved REM sleep on PSG met the criteria for REM sleep without atonia (RSWA) [31]. In a recent case report, a 60-year-old man presented with a 5-year history of REM behavior disorder, followed by rapid-onset constitutional symptoms and neurological decline, and was ultimately diagnosed with CJD. This case sheds light on the potential early REM sleep disturbances in CJD [33]. While RBD has not been established as a primary symptom of CJD, evidence of this disorder preceding the diagnosis of CJD or accompanying the disease progression has been reported in the literature [30].

Currently, there are no disease-modifying treatments available for CJD, although there are several treatments at different phases of pre-clinical and clinical trials. Identifying early subtle signs of CJD in individuals at risk of developing the disease due to their genetic status by combining multiple biomarkers (fluid biomarkers, advanced imaging, and electrophysiological biomarkers) will allow for better risk stratification of conversion into disease. This in turn will enable intervention in the future, at the prodromal stage, before symptoms’ onset. A better understanding of the trajectory of the disease will enable us to identify the window of opportunity for treatment before significant neurodegeneration has taken place. Risk stratification for mutation carriers will also allow a personalized-based approach regarding inclusion in interventional clinical trials, the timing of medical treatment (when it becomes available), and better consultation regarding lifestyle modifications and prognosis. Establishing a well-defined, large, genetically homogeneous cohort of healthy carriers of the E200K mutation (at-risk population for gCJD) in a longitudinal design with repeated collections of multiple biomarkers will enable this risk stratification, and assessment of the safety and efficacy of future treatments.

We established a longitudinal natural history study of first-degree relatives of gCJD with the following aims: To identify biological markers of preclinical CJD and to identify risk factors for phenoconversion. To achieve these aims we collect extensive longitudinal clinical, imaging, and neurophysiological data as well as body fluid and tissue-based biomarkers from healthy and at-risk individuals.

## Design and methods

### Study aims

To identify biological markers of preclinical CJD and risk factors for phenoconversion.

### Study design

The study has two main groups: A group of patients diagnosed with CJD, who are carriers of E200K mutation in the *PRNP* gene (gCJD), and a group of first-degree healthy relatives (HR) (both carriers and non-carriers of the E200K mutation in the *PRNP* gene) of patients diagnosed with gCJD.

**Fig. 1:**
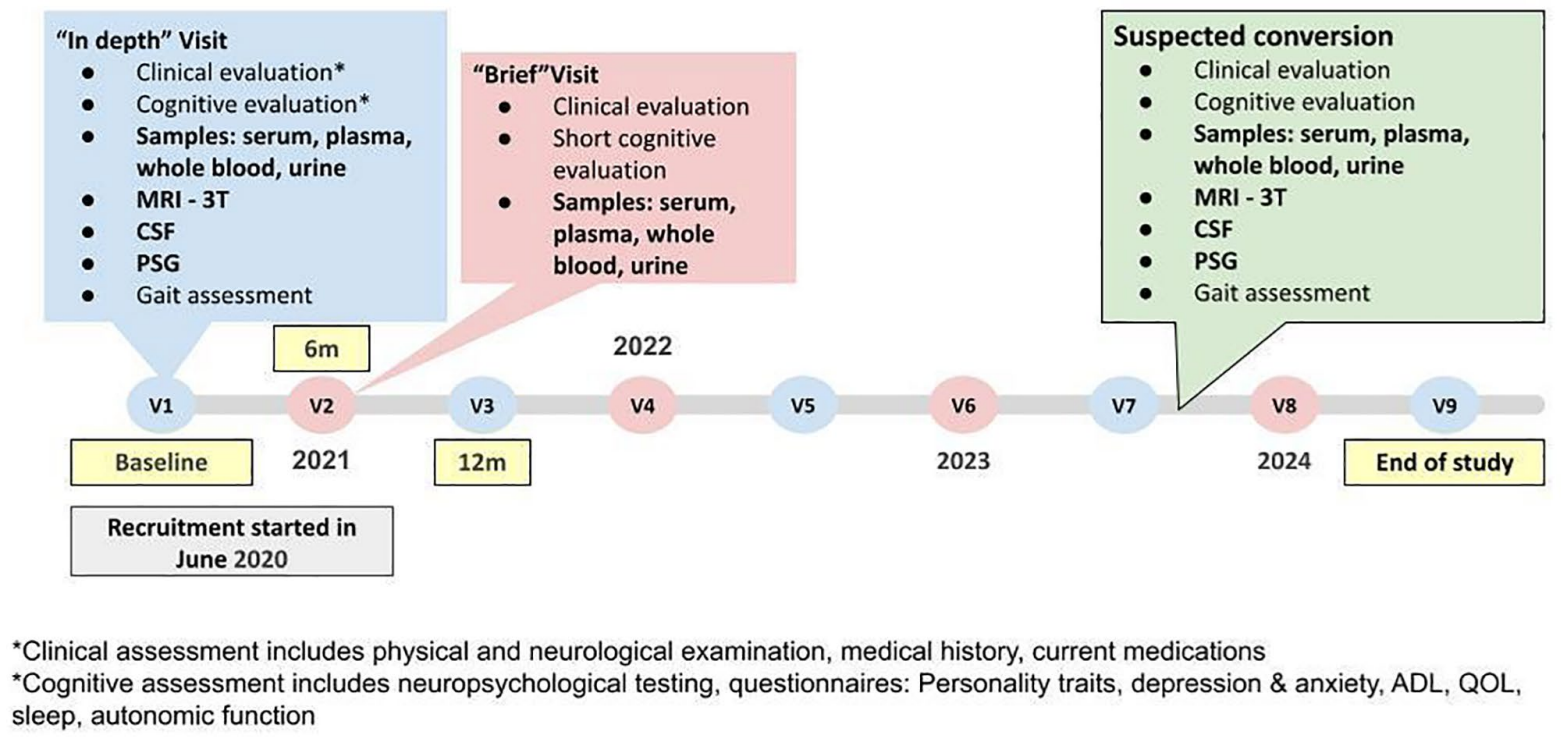
Study design

At baseline, and at the end of every year, participants are invited for an “in-depth” visit, which includes a clinical evaluation, blood and urine collection, gait assessment, brain MRI, lumbar puncture (LP), and PSG. At 6 months from baseline, and then halfway through each year, participants are invited for a “brief” visit, which includes a clinical evaluation, short cognitive assessment, and blood and urine collection (Fig. 1). Participants diagnosed with gCJD will be invited for an extensive visit that encompasses a clinical evaluation, blood and urine collection, gait analysis, brain MRI, LP, and PSG. Given the progressive nature of the disease, patients will only undergo the assessments they can complete. The comprehensive schedule of activities for HR and gCJD patients has been detailed in the supplementary material (Table S1, S2) .

After obtaining informed consent, participants undergo a physical and neurological evaluation performed by a certified neurologist. The participant’s medical and family history will be reviewed as well as his or her concomitant medications. An experienced clinical researcher will then perform the cognitive and gait evaluations and will help participants fill in several questionnaires. The participant is fitted with a gait home-monitoring device and will have direct contact with the study personnel. HR will be encouraged to contact the investigators if any symptoms or medical issues emerge between visits. All Participants will then be scheduled for LP, MRI, and PSG tests. These procedures will be performed on different days but all within 90 days (preferably within 30 days) from the clinical assessment and the provision of the biological samples.

### Study population

Inclusion/ exclusion criteria.

gCJD patients’ inclusion criteria:

- Diagnosis of CJD at baseline/ screening.
- Age 18 years or older at baseline.
- Confirmation of E200K mutation or willingness to undergo genetic testing as part of the pre-screening for new patients.
- Ability to provide written informed consent under Good Clinical Practice (GCP), International Conference on Harmonization (ICH), and local regulations, or having a person with a power of attorney willing to provide written informed consent.

gCJD patients’ exclusion criteria:

- Any other medical or psychiatric condition or laboratory abnormality, which in the investigator’s opinion might preclude participation.
- Previously obtained MRI scan with evidence of clinically significant neurological disorder other than CJD.
- Current anticoagulant treatment (e.g. Non-vitamin K Antagonist Oral Anticoagulants (NOACs), Warfarin, Low Molecular weight Heparin) that might preclude safe completion of LP.
- Conditions that preclude the safe performance of LP, such as severe lumbar spinal disease, bleeding diathesis, or clinically significant coagulopathy or thrombocytopenia.
- Conditions that preclude the safe performance of MRI scannings such as subjects who have a pacemaker, aneurysm clips, artificial heart valves, ear implants, metal fragments or foreign objects in the eyes, skin, or body, or any other known contra-indication for MRI.
- Active malignant disease.

Healthy relatives (HR) inclusion criteria:

- First–degree relative of an E200K gCJD patient.
- Age 50 years or older at baseline.
- Willingness to undergo genetic testing.
- Ability to provide written informed consent under GCP, ICH, and local regulations.
- Willingness and ability to comply with scheduled visits, required study procedures, and laboratory tests.

Healthy relatives’ exclusion criteria include a clinical diagnosis of CJD. Otherwise, exclusion criteria are the same as those described for gCJD patients.

### Recruitment

gCJD patients will be recruited from the CJD clinic or the neurological department at the Tel Aviv “Sourasky” Medical Center (TASMC). Patients who are willing to participate will receive genetic counseling before signing informed consent. They will then be genotyped. Once their genotype is known they will be invited to participate in the study. HR of those patients will be invited to participate in the study if they fulfill the inclusion criteria. All potential participants will be approached by a neurologist who will explain the study procedures, the purpose, potential risks, and benefits of this study and informed consent will be obtained. TASMC serves as a hub for individuals who are suspected or diagnosed with gCJD, making it likely that the majority of gCJD patients in Israel are referred to TASMC. Additionally, Israel has maintained an official registry for all diagnosed gCJD patients since 1965. Patients who decline participation in the study but are seen at TASMC will still be documented in the registry.

### Sample size calculation and statistical analysis

With the objective of identifying biological markers for early detection of gCJD, we intend to conduct a comparative analysis of marker levels between gCJD patients and healthy individuals, as well as between healthy carriers and non-carriers. Based on the Israel National Registry for CJD, the annual incidence rate of CJD is 51 cases, although this is believed to be an underestimation. About two-thirds of the reported cases are familial cases, and of these cases, almost all are carriers of the E200K mutation. Given our conservative estimate that 50% of new cases of gCJD will be referred to our center, we anticipate that we will receive 15 new gCJD patients each year, for a total of 60 patients during the study period. Furthermore, we anticipate that approximately one-third of these patients, or 20 in total, will be willing to participate in the study.

With a conservative estimation of 3 first-degree HR per gCJD patient, this enables a population pool of > 100 per study period. As the E200K is an autosomal dominant mutation, 50% of the first-degree relatives of gCJD will carry the mutation. Based on Spudich et al. [34], the likelihood of developing gCJD is 0.45 in the age group of 51–60. According to Minikel et al., the average annual probability of onset (phenoconversion) for healthy carriers aged 40 to 80 years is 4.6% [35]. The primary aim of the study relates to biological markers for identifying phenoconversion. In our study, we referred to a positive CSF RT-QuIC.

CSF RT-QuIC has proven to be a relatively sensitive (73-97%) [13, 14] and a highly specific biomarker (99-100%) [12, 15–17] for diagnosing CJD, and it is now part of the standard diagnostic criteria [10]. Thus, considering a conservative estimate of a 13.6% phenoconversion rate among carriers (calculated as 3.4% expected conversion per year for 4 years) a sample size of 106 healthy relatives (53 carriers and 53 non-carriers) is required, based on a desired probability of type 1 error of 0.05 and a power of 80% (https://clincalc.com/stats/samplesize.aspx).

Secondary outcomes relating to the risk of phenoconversion will be also examined. With the objective of identifying risk factors for phenoconversion, we intend to conduct a comparison between groups (gCJD patients vs. HR), and within the HR group (carriers vs. non-carriers). We also intend to perform a Cox proportional hazards analysis. The analysis is based on the concept of hazard, which is a measure of the instantaneous risk of the event occurring at a given time. The Cox model assumes that the hazard for each individual is proportional to the hazard for others over time, and can be represented as a combination of individual-specific factors and their coefficients. The coefficients from the Cox model can be used to estimate the relative risk of phenoconversion for each factor, with larger coefficients indicating a greater risk of disease onset. By examining the relationship between the risk factors and phenoconversion time, Cox proportional hazards analysis can help identify which factors are most important in determining the risk of disease onset, allowing for the development of predictive models and the identification of potential targets for intervention.

### Data management

Data Collection: All data collected during the study will be recorded electronically and stored on secure, encrypted servers. All study participants will be assigned a unique identifier to maintain confidentiality and to link data from different visits and assessments. Participant retention: All individuals participating in the study will be provided with a comprehensive report of the clinical assessments performed, which include physical and neurological examinations, MRI results, PSG clinical report, LP clinical results (cell count, protein and glucose levels), biomic blood tests, and cognitive assessments. In accordance with the participants’ preferences, the Principal Investigator (PI) will communicate all significant clinical data to their family physician, thereby promoting preventive medicine and the overall health of the participants. Additionally, annual webinars will be arranged to update the participants about the progress of the study and advancements in the related research field. Contact information for both the PI and the study coordinator will be made available to the participants, who will be encouraged to reach out to the study personnel with any questions or concerns.

Data Quality Control: To ensure data quality, a trained research assistant will perform regular data entry checks, and all data will be reviewed by the principal investigator to identify and resolve any discrepancies. Data monitoring: The local and the Ministry Of Health (MOH) IRB’s are responsible for conducting periodic audits on the conduct of the trial. The study meets all of the ethical and regulatory requirements for data collection and use and does not require additional oversight. Data Backup: Daily backups of all study data will be stored off-site, in a secure location, to ensure data integrity and protect against loss or corruption. Data Access: Only authorized personnel, who have completed confidentiality agreements, will have access to the study data. All study personnel will be trained on data security protocols. Data Analysis: Statistical analysis of the data will be performed using appropriate software. Data will be examined for normalcy and homogeneity based on scatter plots and histograms. Differences between groups on clinical and biological measures will be performed using General Linear Models for repeated measures. Estimation of risk based on aggregated measures will be done using cox regression. Data Sharing: The study data will be shared with relevant researchers and institutions in accordance with the study’s approved data-sharing plan. All data sharing will be done in a secure and confidential manner, and in compliance with relevant privacy regulations. Communication of trial results will be done via publication in scientific journals and professional conventions, or other data-sharing arrangements. Data Archiving: All study data will be archived and stored in a secure location for a minimum of 10 years after the completion of the study. Trial registration: The study is an observational study. It has registered retrospectively in https://clinicaltrials.gov/ and has been assigned an identification number NCT05746715.

## Methods

### Clinical neurological and cognitive evaluation

Healthy relatives of CJD patients will undergo the following longitudinal assessments:

- Collection of clinical data, including cognitive, behavioral, and autonomic function.
- Collection of Blood, urine, and CSF biomarkers.
- Electrophysiological measurements and assessment of other sleep characteristics.
- structural and functional markers on brain MRI.
- Gait and movement assessments.

Data regarding demographics, medical and family history as well as physical and neurological examinations will be collected on all participants. Participants will be asked to provide information as to food and drink consumption on the day of the exam. Concomitant medications, including over-the-counter dietary supplements (e.g., herbal remedies), or prescription medications, will be recorded. Evaluation of individuals who take antibiotics will be deferred until their course of antibiotics ends, with a washout period of at least 1 week.

The justification for the need for LP in this study is based on several factors. Firstly, CJD is a rapidly progressive disease, and early detection is critical for improving patient outcomes. The yearly analysis of the CSF may help identify prodromal changes in brain biomarkers, particularly the aggregation of PrP^sc^, which may indicate onset of disease before appearance of clinical symptoms. Secondly, the protocol has been granted approval by the ethics committee, taking into account the exceptionally high penetrance of the mutation and the valuable data that can only be obtained from CSF. This taken together with the fact that the LP procedure is considered safe led the committee to decide that its potential benefit outweighs the risk. Thirdly, participants who are not willing to repeat the LP procedure are not excluded from the study. However, in the event that they exhibit symptoms suggestive of conversion into the disease, an LP is carried out as part of the clinical diagnostic process. This ensures that participants are given the option to opt-out of the LP procedure while still receiving the necessary medical attention should they develop symptoms. In conclusion, there is a need for lumbar puncture in healthy carriers of the E200K mutation for CJD as part of a clinical trial studying the natural history of the disease and the prodromal phase. CSF sampling in a high risk healthy population is critical in order to identify markers associated with the prodromal phase of the disease and therefore was approved by the ethics committee. Participants who do not wish to undergo LP are not excluded and are able to participate in the study.

All gCJD patients will be scored according to the National Prion Monitoring Cohort (NPMC) Scale (MRC Prion Disease Rating Scale) [36], which is a multimodal scale assessing both cognitive impairment and daily function disability. This scale consists of 11 parameters: bowel function, bladder function, toilet use, bathing, feeding, transfer and mobility, stairs, best verbal response, memory and orientation to surroundings, judgment and problem-solving, and use of tools. Each section is scored between 0 and 4 with 0 indicating complete disability and 4 normal ability, for a maximum of 20 points.

### Cognitive assessment

Standardized neuropsychological tests assessing different cognitive domains will provide an in-depth evaluation of cognitive function. These tests include the following:

- Montreal Cognitive Assessment (MoCA) [37] - A 30-point cognitive screening instrument that assesses visuospatial, executive, naming, attention, language, abstraction, delayed recall, and orientation domains.
- Verbal Fluency tests [38] on which participants name as many different words that begin with a designated letter or as many items that belong to a given semantic category in one minute. The score is the sum of all correct words produced.
- The Motor-free Visual Perception Test (MVPT-3) [39], which measures the ability to discriminate dominant features of different objects, including the ability to discriminate position, shapes, and forms, and the ability to perceive the positions of objects in relation to oneself and other objects. This test also examines visual memory, the ability to distinguish an object from the surrounding objects, and the ability to perceive a whole figure from its fragments.
- The Frontal Assessment Battery (FAB) [40] targets executive functions, including attention span, working memory, motor planning, abstraction, and inhibition.
- Digit span (Wechsler Adult Intelligence Scale, Third Edition, WAIS-III) which measures verbal working memory by asking participants to repeat digits forward and backward.
- The Cookie theft picture description from the Boston Diagnostic Aphasia Examination–Third Edition (BDAE).
- Trail Making Test (TMT) parts A & B [41] which estimates visual search, speed of processing, and shifting attention.

### Affect, behavior, and habits

The following questionnaires assess the level of anxiety, depression, stress, and personality traits:

- The Beck Depression Inventory (BDI) [42] which evaluates the presence and intensity of depression.
- The State-Trait Anxiety Inventory (STAI) [43] which measures emotional state anxiety, using 40 items.
- The Social Readjustment Rating Scale (SRRS) [44] aims to identify major stressful life events.
- The Big-5 Questionnaire [45] measures dimensions of personality through ratings of 44 items.

### Autonomic function

Questionnaires assessing autonomic function and sleep will be completed:

- The Epworth Sleepiness Scale (ESS), a self-administered questionnaire collecting information on the propensity to fall asleep in eight different situations encountered commonly in daily life. Each situation is rated from 0 (no chance of dozing) to 3 (high probability of dozing), and the total score ranges from 0 to 24. Total scores of zero to 10 are normal, 10–12 are borderline, and scores from 12 to 24 are abnormal.
- The REM Sleep Behavior Disorder Screening Questionnaire (RBDSQ) is a 10-item self-rated questionnaire to assess sleep-wake disturbances. Sleep behavior disorder may represent early manifestations of progressive neurodegenerative disorders, including Parkinson’s disease. A score greater than 5 reflects RBD.
- SCOPA-AUT (Scales for Outcomes in Parkinson’s disease - Autonomic Dysfunction) - The SCOPA-AUT was developed to evaluate autonomic symptoms in patients with Parkinson’s disease as well as patients with Multiple System Atrophy. The scale is self-completed by patients and consists of 25 items assessing the following domains: gastrointestinal (7 items), urinary (6 items), cardiovascular (3 items), thermoregulatory (4 items), pupillomotor (1 item), and sexual (2 items for men and 2 items for women).

### Blood and urine samples

#### Samples handling and pre-processing

Participants will provide blood and urine samples when clinically assessed. They will be strongly encouraged to eat a low-lipid diet on the day of the test. Information on food intake will be collected. Samples will be delivered to the laboratory as soon as possible, and not more than 30 min after collection. Processing and extractions will occur immediately after the arrival of samples in the laboratory.

Serum extraction: To ensure serum clotting, the serum tube will be centrifuged at least 30 min after blood drawing (and no longer than 60 min) at 1500 g for 15 min at 4°c. Then, on ice, the supernatant will be transferred to a conical 15ml tube and then inverted gently. The supernatant will be evenly divided into four aliquots and frozen at -80°c.

Plasma extraction (K2EDTA tube): Tubes will be centrifuged for no longer than 30 min after blood drawing at 2000 g for 15 min at 25°c. The supernatant will be transferred to a conical 15ml tube and then inverted gently. Plasma will be divided into 8 aliquots of 0.5ml and frozen at -80°c. Buffy Coat (BC, K2EDTA tube): Immediately after transferring the plasma from the K2EDTA tube, BC will be cautiously collected to a labeled cryotube at RT and frozen at -80°c.

Urine processing: Urine tubes will be centrifuged upon arrival at 2500 g for 15 min at 4°c. Then, on ice, the supernatant will be transferred to a conical 15ml tube and then inverted gently. The urine will be divided between two conical 15ml tubes, 4ml to the first tube and the remainder to the second. If the total amount of urine is less than 5ml, the urine will be divided evenly between the tubes. The tubes will be frozen at -80°c.

Whole blood tubes will be frozen no longer than 60 min after the blood draw.

PAX tubes will be collected for future RNA extraction. The tubes will be kept at room temperature for 3–26 h and then frozen at -80°c. Whole blood for DNA extraction will be collected only at the first visit. The tube will be placed at 4°c until DNA is extracted.

Sample data will be inserted into a dedicated database software.

#### Genetic testing

Genomic DNA will be extracted from whole blood samples and will be genotyped for the PRNP-E200K mutation (rs28933385, TaqMan genotyping assay C_27531205_10_F_; Applied Biosystems, Foster City, CA, USA). Confirmation for the *PRNP* mutation will be done by a polymerase chain reaction and Sanger Sequencing. Confirmation by sequencing will be performed for healthy relatives that are shown to be E200K carriers and for all CJD patients.

#### Clinical laboratory tests

Blood will be collected for standard clinical laboratory tests: Complete Blood Count, Chemistry screen, Glycohemoglobin test, Vitamin D, Vitamin B12, Thyroid Function Tests, C-reactive protein, Lipid profile, International Normalized Ratio, Prothrombin Time, and Partial Thromboplastin Time.

Other fluid biomarkers in blood will include total-tau (T-tau), phosphorylated-tau (P-tau), Neurofilament light chain (NfL), Glial fibrillary acidic protein (GFAP), and Ubiquitin carboxy-terminal hydrolase L1 (UCHL-1).

#### Lumbar puncture (LP)

LP will be performed by an experienced neurologist at the Neurological Institute at TASMC. An LP for the collection of 15–20 ml of CSF will be carried out on all participants in the longitudinal study at baseline and at the end of every year thereafter, and when phenoconversion is suspected unless there is evidence of clinically significant coagulopathy or thrombocytopenia that would interfere with the safety of the procedure. LP will be performed with an a-traumatic needle (G25 pencil LP, AVMAD). Participants will be monitored during the procedure and following the procedure and will remain under bed rest supervision in the unit for 1.5 h following the procedure. They will be contacted by phone the day after the procedure as well as 7 to 10 days following an LP to monitor any adverse events.

The first 2ml of CSF will be processed to conduct standard analyses on cell count, protein, and glucose levels. The rest of the CSF will be centrifuged at 2000 g for 10 min at 24 °C and separated into 500ml aliquots in 1.5ml tubes, four of them with 0.03% CHAPS. The pellet will be suspended in 1.5ml of RNAlater Solution, placed at 4 °C for approximately 24 h, and stored at -80 °C.

Other fluid biomarkers in CSF will include T-tau, P-tau, NfL, Neurogranin, Neuronal glycolytic enzyme (NSE), GFAP, YKL-40 (also known as Chitinase 3-like 1), UCHL-1, S100B, PrP^sc^, and Mitochondrial malate dehydrogenase 1 (MDH1) levels.

#### Imaging

Participants in the longitudinal study will undergo an MRI brain scan at the baseline visit, 12, 24, 36, and 48 months thereafter, and if phenoconversion is suspected. MRI scans will be performed at the Sagol Brain Institute, using a 3-T Magnetom Prisma (Siemens Healthineers, Erlangen, Germany) with a 20 channels phased-array head coil. MRI protocol will include structural imaging including T1 and T2-FLAIR weighted imaging to assess brain structure and pathology; T1, T2, and T2* relaxometry, to quantitatively assess the tissue relaxation times Diffusion Tensor Imaging (DTI) to assess tissue maturation and structural connectivity; resting-state fMRI – to assess functional connectivity. The imaging parameters of each sequence are given in Table 1.

**Table 1:** MR Imaging parameters

Sequence	TR ( Repetition time) [ms ± SD]	TE (Echo time) [ms ± SD]	In-Plan Resolution (XxY) [mm^2^]	Slice Thickness [mm]	Number of Slices	Scanning time (min)
T2-FLAIR	8000	117	0.8 × 0.8	5	32	2.58
T1-MP2TAGE	5000	3.43	1 × 1	1	176	7.07
DTI^+^	7200	55	1.8 × 1.8	1.8	76	7.57
rsfMRI	2500	30	2.3 × 2.3	3	42	6.08
T2*	1350	^++^2.8–80 (8 TEs)	0.9 × 0.9	3	32	6.18
T1-MPRAGE	2200	3.22	1 × 1	1	192	5.06
T2-map	2670	^+++^17.5–105 (6 TEs)	0.6 × 0.6	3	24	6.59
^++++^T1-IR	2500	12	0.7 × 0.7	4	24	4.02

^+^30 directions, with 3 b-values: 0, 500 and 1000 s/mm

^++^TEs = 12.81, 11.68, 23.07, 34.46, 45.85, 57.24, 68.63, 80.00 ms

^+++^TEs = 17.5, 35.0, 52.5, 70.0, 87.5, 105.0 ms

^++++^With inversion time = 500 ms

**Table 1:** Imaging parameters

Sequence	TR ( Repetition time) [ms ± SD]	TE (Echo time) [ms ± SD]	In-Plan Resolution (XxY) [mm^2^]	Slice Thickness [mm]	Number of Slices	Scanning time (min)
T2-FLAIR	8000	117	0.8 × 0.8	5	32	2.58
T1-MP2TAGE	5000	3.43	1 × 1	1	176	7.07
DTI^+^	7200	55	1.8 × 1.8	1.8	76	7.57
rsfMRI	2500	30	2.3 × 2.3	3	42	6.08
T2*	1350	^++^2.8–80 (8 TEs)	0.9 × 0.9	3	32	6.18
T1-MPRAGE	2200	3.22	1 × 1	1	192	5.06
T2-map	2670	^+++^17.5–105 (6 TEs)	0.6 × 0.6	3	24	6.59
^++++^T1-IR	2500	12	0.7 × 0.7	4	24	4.02

^+^30 directions, with 3 b-values: 0, 500 and 1000 s/mm

^++^TEs = 12.81, 11.68, 23.07, 34.46, 45.85, 57.24, 68.63, 80.00 ms

^+++^TEs = 17.5, 35.0, 52.5, 70.0, 87.5, 105.0 ms

^++++^With inversion time = 500 ms

#### PSG, EEG monitoring, and physiological measurements during sleep

PSG includes EEG monitoring combined with physiological measurements during wakefulness and throughout overnight sleep as follows. High-density EEG is recorded continuously using a 256-channel hydrocel geodesic sensor net (Magstim Electrical Geodesics, Inc. system [Magstim-EGI]). Each carbon-fiber electrode consists of a silver-chloride carbon fiber pellet, a lead wire, and a gold-plated pin, and is injected with conductive gel (Electro-Cap International). Signals are referenced to Cz, amplified via an AC-coupled high-input impedance amplifier (NetAmps 300, EGI), and digitized at 1000 Hz. Electrode impedance in all sensors is verified to be < 50 kΩ before starting the recording. EEG is band-pass filtered offline between 0.5 and 45 Hz, and an additional notch filter at 50 Hz is applied to the continuous data offline to remove residual line noise.

Beyond EEG, additional physiological signals include synchronized video, electrooculogram (EOG), electromyogram (EMG), heart rate measurements via chest electrodes, breathing assessment including SPO2/saturation, nasal airflow, and belts monitoring chest and abdomen efforts.

#### Gait rhythmicity, arm swing, and axial rotation

Participants will be asked to perform motor tasks in the clinic while wearing lightweight accelerometers on their lower back and wrists. A total of 3 tasks will be administered:

- Sway: Participants will be asked to stand with their feet together for 30 s while their eyes are open and then for 30 s while their eyes are closed to assess sway and subtle changes in stability. Velocity, direction, jerk, and displacement of the center of mass during the task will be determined.
- Gait: Participants will be asked to walk for one minute under two different walking conditions: comfortable speed and walking while performing a cognitive task of serial subtraction of 7 from a predefined 3-digit number. Swing time, average stride time, stride and swing time variability, and magnitude and symmetry of arm swing and axial rotation will be determined.
- The Timed Up and Go (TUG): This performance-based measure evaluates sit-to-stand transfer, walking for 3 m, turning, and coming back to sitting on a chair. The TUG will be performed twice.

These validated tests provide information on fall risk (time of performance), transfer abilities, turning, and ability to follow instructions.

Ambulation and activity at home and in the community will be assessed using a body-worn sensor. Mobility quantity and quality (e.g., number of steps taken over 7 days, gait quality) will be measured using a small, lightweight, waterproof body-fixed sensor (i.e., accelerometer) worn on the back for 7 consecutive days following the assessment in the clinic. The device (AX3, Axivity Ltd, UK) allows for objective determination of the amount and type of activity performed by the participant. This, for example, includes time spent lying, sitting, standing, and walking and the quality of each of these activities over every 7 days. These measures reflect different aspects of health status and are also related to disease outcomes. In addition, the quality of mobility will be assessed by evaluating step time, variability, and rhythmicity of movement. The device will be returned to the clinic after 7 days via courier service.

## Discussion

This project aims to identify biological markers of preclinical CJD and risk factors for phenoconversion. The relatively large and homogeneous cohort of healthy carriers of the E200K mutation, as well as the longitudinal design and comprehensive protocol, should allow for better characterization of the preclinical phase of gCJD. The prospective clinical and behavioral data may also help to identify environmental risk factors for phenoconversion. The long follow-up increases the possibility of phenoconversion during the study period, which is crucial for the identification of both early markers of disease and risk factors. In other neurodegenerative diseases, such as Parkinson’s disease, validated criteria for the diagnosis of early or prodromal disease exist [46]. The results from this study may contribute to the establishment of similar criteria for prodromal CJD and identifying the “window of opportunity” for preventive interventions.

There are several disease-modifying drugs under development in different phases of pre-clinical and clinical trials. These treatments will likely need to be given at the very early stages of the disease before significant neurodegeneration occurs, or even to healthy individuals at-risk for phenoconversion.

The continuous follow-up of the participants and the frequent assessments (every 6 months for 4 years) will allow early identification and diagnosis in case of conversion into disease. Identifying gCJD patients at the very early clinical or even prodromal phase could also facilitate their enrollment in future clinical trials for disease-modifying drugs. The knowledge generated from this study is likely to advance the understanding of the underlying clinicopathological processes that occur at the very beginning of CJD, as well as potential genetic and environmental risk factors for the development of the disease, therefore advancing the development of safe and efficient interventions.

## Electronic supplementary material

Below is the link to the electronic supplementary material.

Supplementary Material 1

Supplementary Material 2

## Data Availability

Data regarding the study protocol presented here are detailed in the main manuscript or additional supporting files.

## List of abbreviations

CJD: Creutzfeldt-Jakob Disease
PrP: Prion protein
PrP^c^: Normal prion protein
PrP^sc^: Pathological prion protein
sCJD: Sporadic Creutzfeldt-Jakob Disease
gCJD: Genetic Creutzfeldt-Jakob Disease
CSF: Cerebrospinal fluid
MRI: Magnetic resonance imaging
EEG: Electroencephalography
RT-QuIC: Real-time quaking-induced conversion
NF: Neurofilaments
REM: Rapid eye movement
RBD: Rapid eye movement sleep behavior disorder
PSG: Polysomnography
RSWA: Rapid eye movement sleep without atonia
HR: Healthy relatives
LP: Lumbar puncture
GCP: Good clinical practice
ICH: International Conference on Harmonization
NOACs: Non-vitamin K anticoagulants
TASMC: Tel Aviv “Sourasky” Medical Center
NPMC: National Prion Monitoring Cohort
MRC scale: Monitoring cohort disease rating scale
MoCA: Montreal Cognitive Assessment
MVPT-3: Motor-free Visual Perception Test
FAB: Frontal Assessment Battery
WAIS-III: Wechsler Adult Intelligence Scale, Third Edition
BDAE: Boston Diagnostic Aphasia Examination–Third Edition
TMT: Trail Making Test
BDI: Beck Depression Inventory
STAI: State-Trait Anxiety Inventory
SRRS: Social Readjustment Rating Scale
ESS: Epworth Sleepiness Scale
RBDSQ: REM Sleep Behavior Disorder Screening Questionnaire
SCOPA-AUT: Scales for Outcomes in Parkinson’s disease - Autonomic Dysfunction
BC: Buffy Coat
T-tau: Total Tau
P-tau: Hyperphosphorylated Tau
NfL: Neurofilament light chain
GFAP: Glial fibrillary acidic protein
UCHL-1: Ubiquitin carboxy-terminal hydrolase L1
NSE: Neuronal glycolytic enzyme
MDH1: Mitochondrial malate dehydrogenase 1
EOG: Electrooculogram
EMG: Electromyogram
TUG: Timed Up and Go
