# Correction: The natural history study of preclinical genetic Creutzfeldt-Jakob Disease (CJD): a prospective longitudinal study protocol

**DOI:** 10.1186/s12883-023-03272-w

**Published:** 2023-06-13

**Authors:** Noa Bregman, Tamara Shiner, Gitit Kavé, Roy Alcalay, Mali Gana-Weisz, Orly Goldstein, Tal Glinka, Orna Aizenstein, Dafna Ben Bashat, Yifat Alcalay, Anat Mirelman, Avner Thaler, Nir Giladi, Nurit Omer

**Affiliations:** 1 Cognitive Neurology Unit, Neurological Institute, Tel-Aviv Medical Center, Tel-Aviv, Israel; 2 Sackler School of Medicine, Tel-Aviv University, Tel-Aviv, Israel; 3 Sagol School of Neuroscience, Tel-Aviv University, Tel-Aviv, Israel; 4 Department of Education and Psychology, The Open University, Ra’anana, Israel; 5 Laboratory of biomarkers and genomic of neurodegeneration, Tel-Aviv Medical Center, Tel-Aviv, Israel; 6 Sagol Brain Institute, Wohl Institute for Advanced Imaging, Sourasky Medical Center, Tel Aviv, Israel; 7 Department of Diagnostic Imaging, Sourasky Medical Center, Tel Aviv, Israel; 8 Division of Clinical Laboratories, Tel Aviv Sourasky Medical Center, Tel- Aviv, Israel; 9 Laboratory of early markers of neurodegeneration, Neurological Institute, Tel-Aviv Sourasky Medical Center, Tel-Aviv, Israel; 10 Brain Institute, Tel Aviv Sourasky Medical Center, Tel Aviv, Israel

**Correction: BMC Neurol 23, 151 (2023)**

10.1186/s12883-023-03193-8

Following publication of the original article [1], the authors identified an error to the presentation of the author names. The given names and family names for all authors were erroneously transposed.

The author group has been updated above and the original article [1] has been corrected.
