# Supplementary material for: The natural history study of preclinical genetic Creutzfeldt-Jakob Disease (CJD): a prospective longitudinal study protocol
Source: BMC Neurol. 2023 Apr 14;23:151. doi: 10.1186/s12883-023-03193-8 (PMC10108539; doi:10.1186/s12883-023-03193-8)
Supplement: Supplementary file 1 — Supplementary Material 1 [file 12883_2023_3193_MOESM1_ESM.docx]

Table S1: Schedule of activities for healthy relatives:

| **Visit Number** |  | **V1 (Baseline)** | **V2** | **V3** | **V4** | **V5** | **V6** | **V7** | **V8** | **V9** | **Unscheduled Visit (Suspected Phenoconvertion)** |
| --- | --- | --- | --- | --- | --- | --- | --- | --- | --- | --- | --- |
| **Months/  Visit description** |  | **0** | **6** | **12** | **18** | **24** | **30** | **36** | **42** | **48** |  |
| Consent forms | Written Informed Consent (genetic and clinical) | X |  |  |  |  |  |  |  |  |  |
| Medical | Medical History/Demographics | X | X | X | X | X | X | X | X | X | X |
|  | Family Medical History | X | X | X | X | X | X | X | X | X | X |
|  | Physical Examination | X | X | X | X | X | X | X | X | X | X |
|  | Neurological Examination | X | X | X | X | X | X | X | X | X | X |
|  | Vital Signs | X | X | X | X | X | X | X | X | X | X |
|  | Concomitant Medication | X | X | X | X | X | X | X | X | X | X |
| Blood and Urine Sampling | Blood Sample for DNA | X | X | X | X | X | X | X | X | X | X |
|  | Clinical Laboratory Assessments | X | X | X | X | X | X | X | X | X | X |
|  | Bio-Fluid collection (Blood and Urine) | X | X | X | X | X | X | X | X | X | X |
| Cognitive Function | Verbal fluency | X |  | X |  | X |  | X |  | X | X |
|  | MVPT | X |  | X |  | X |  | X |  | X | X |
|  | FAB | X |  | X |  | X |  | X |  | X | X |
|  | Digit span | X |  | X |  | X |  | X |  | X | X |
|  | TMT | X |  | X |  | X |  | X |  | X | X |
|  | Montreal Cognitive Assessment (MoCA) | X |  | X |  | X |  | X |  | X | X |
|  | Cookie theft picture | X |  | X |  | X |  | X |  | X | X |
| Autonomic Function | Epworth Sleepiness Scale | X |  | X |  | X |  | X |  | X | X |
|  | REM Sleep Behavior Questionnaire | X |  | X |  | X |  | X |  | X | X |
|  | SCOPA-AUT | X |  | X |  | X |  | X |  | X | X |
| Behaviour, habits, and affect | State-Trait Anxiety Inventory for Adults | X |  | X |  | X |  | X |  | X | X |
|  | Beck's Depression Inventory (BDI) | X |  | X |  | X |  | X |  | X | X |
|  | SRRS | X |  | X |  | X |  | X |  | X | X |
|  | Big-5 | X |  | X |  | X |  | X |  | X | X |
| Gait Assessment | Laboratory Gait assessment | X |  |  | X |  | X |  | X |  | X |
|  | Home monitoring (wearable sensors) | X |  |  | X |  | X |  | X |  | X |
| Sleep Assessment | Polysomnography (PSG) | X |  | X |  | X |  | X |  | X | X |
| CSF sampling | Lumbar puncture | X |  | X |  | X |  | X |  | X | X |
| Brain Imaging | MRI scan | X |  | X |  | X |  | X |  | X | X |

Table S2: Schedule of activities for gCJD patients:

| **Visit Number** |  | **V1** |
| --- | --- | --- |
| **Months/  Visit description** |  | **0** |
| Consent forms | Written Informed Consent (genetic and clinical) | X |
| Medical | Medical History/Demographics | X |
|  | Family Medical History |  |
|  | Physical Examination | X |
|  | Neurological Examination | X |
|  | Vital Signs | X |
|  | Concomitant Medication | X |
|  | MRS score | X |
| Blood and Urine Sampling | Blood Sample for DNA | X |
|  | Clinical Laboratory Assessments | X |
|  | Bio-Fluid collection (Blood and Urine) | X |
| Cognitive Function | Verbal fluency | X |
|  | MVPT | X |
|  | FAB | X |
|  | Digit span | X |
|  | TMT | X |
|  | Montreal Cognitive Assessment (MoCA) | X |
|  | Cookie theft picture | X |
| Autonomic Function | Epworth Sleepiness Scale | X |
|  | REM Sleep Behavior Questionnaire | X |
|  | SCOPA-AUT | X |
| Behaviour, habits, and affect | State-Trait Anxiety Inventory for Adults | X |
|  | Beck's Depression Inventory (BDI) | X |
|  | SRRS | X |
|  | Big-5 | X |
| Gait Assessment | Laboratory Gait assessment | X |
|  | Home monitoring (wearable sensors) | X |
| Sleep Assessment | Polysomnography (PSG) | X |
| CSF sampling | Lumbar puncture | X |
| Brain Imaging | MRI scan | X |
