# Supplementary material for: The natural history study of preclinical genetic Creutzfeldt-Jakob Disease (CJD): a prospective longitudinal study protocol
Source: BMC Neurol. 2023 Apr 14;23:151. doi: 10.1186/s12883-023-03193-8 (PMC10108539; doi:10.1186/s12883-023-03193-8)
Supplement: Supplementary file 2 — Supplementary Material 2 [file 12883_2023_3193_MOESM2_ESM.docx]

**Supplementary table: SPIRIT checklist**

| Section/item | Item No | Description | Comment | Page No |
| --- | --- | --- | --- | --- |
| **Administrative information** | | |  |  |
| Title | 1 | Descriptive title identifying the study design, population, interventions, and, if applicable, trial acronym | We have changed the title to be more descriptive: *“A natural history study of preclinical genetic Creutzfeldt-Jakob Disease (CJD): A longitudinal observational clinical, imaging and biomarker study protocol”* | 1 |
| Trial registration | 2a | Trial identifier and registry name. If not yet registered, name of intended registry | We added a **Trial registration** section to the abstract and manuscript stating that this protocol was registered retrospectively as an observational study, on February 15th 2023, NCT05746715 | 2,11-12 |
|  | 2b | All items from the World Health Organization Trial Registration Data Set | NA (observational study) |  |
| Protocol version | 3 | Date and version identifier | We have now added the date and version identifier of the protocol in the “Ethics approval and consent” section, under the “Declarations” section. | 23 |
| Funding | 4 | Sources and types of financial, material, and other support | The financial funding of Ionis Pharmaceuticals is mentioned in the “Funding” section, under the “Declarations” section | 23 |
| Roles and responsibilities | 5a | Names, affiliations, and roles of protocol contributors | We have now added this information to the “author’s contribution” section under the “Declarations” section | 23 |
|  | 5b | Name and contact information for the trial sponsor | We have now added the contact information for the trial sponsor in the “funding” section, under the “declarations” section | 23 |
|  | 5c | Role of study sponsor and funders, if any, in study design; collection, management, analysis, and interpretation of data; writing of the report; and the decision to submit the report for publication, including whether they will have ultimate authority over any of these activities | We have now added the following to the the “funding” section, under the “declarations” section: *“Ionis Pharmaceuticals is not involved in the design of the study, data collection, management, analysis and interpretation, report writing, or decision-making regarding publication submission.”* | 23 |
|  | 5d | Composition, roles, and responsibilities of the coordinating centre, steering committee, endpoint adjudication committee, data management team, and other individuals or groups overseeing the trial, if applicable (see Item 21a for data monitoring committee) | NA (observational study) |  |
| Introduction |  |  | In line with SPIRIT guidelines |  |
| Background and rationale | 6a | Description of research question and justification for undertaking the trial, including summary of relevant studies (published and unpublished) examining benefits and harms for each intervention |  | 4-6 |
|  | 6b | Explanation for choice of comparators |  | 4-6 |
| Objectives | 7 | Specific objectives or hypotheses |  | 7 |
| Trial design | 8 | Description of trial design including type of trial (eg, parallel group, crossover, factorial, single group), allocation ratio, and framework (eg, superiority, equivalence, noninferiority, exploratory) |  | 7 |
| **Methods: Participants, interventions, and outcomes** | | |  |  |
| Study setting | 9 | Description of study settings (eg, community clinic, academic hospital) and list of countries where data will be collected. Reference to where list of study sites can be obtained | We have now added a description of the setting to the “study design section”: “*At baseline, and at the end of every year, participants are invited for an “in-depth” visit at the Cognitive Neurology Unit at Tel-Aviv Medical Center*”. | 7 |
| Eligibility criteria | 10 | Inclusion and exclusion criteria for participants. If applicable, eligibility criteria for study centres and individuals who will perform the interventions (eg, surgeons, psychotherapists) |  | 7-8 |
| Interventions | 11a | Interventions for each group with sufficient detail to allow replication, including how and when they will be administered | NA (observational study) |  |
|  | 11b | Criteria for discontinuing or modifying allocated interventions for a given trial participant (eg, drug dose change in response to harms, participant request, or improving/worsening disease) | NA (observational study) |  |
|  | 11c | Strategies to improve adherence to intervention protocols, and any procedures for monitoring adherence (eg, drug tablet return, laboratory tests) | NA (observational study) |  |
|  | 11d | Relevant concomitant care and interventions that are permitted or prohibited during the trial | NA (observational study) |  |
| Outcomes | 12 | Primary, secondary, and other outcomes, including the specific measurement variable (eg, systolic blood pressure), analysis metric (eg, change from baseline, final value, time to event), method of aggregation (eg, median, proportion), and time point for each outcome. Explanation of the clinical relevance of chosen efficacy and harm outcomes is strongly recommended |  | 9-10 |
| Participant timeline | 13 | Time schedule of enrolment, interventions (including any run-ins and washouts), assessments, and visits for participants. A schematic diagram is highly recommended (see Figure) |  | Figure 1  Page 30 |
| Sample size | 14 | Estimated number of participants needed to achieve study objectives and how it was determined, including clinical and statistical assumptions supporting any sample size calculations |  | 9-10 |
| Recruitment | 15 | Strategies for achieving adequate participant enrolment to reach target sample size |  | 9 |
| **Methods: Assignment of interventions (for controlled trials)** | | | NA (observational study) |  |
| Allocation: |  |  |  |  |
| Sequence generation | 16a | Method of generating the allocation sequence (eg, computer-generated random numbers), and list of any factors for stratification. To reduce predictability of a random sequence, details of any planned restriction (eg, blocking) should be provided in a separate document that is unavailable to those who enrol participants or assign interventions | NA (observational study) |  |
| Allocation concealment mechanism | 16b | Mechanism of implementing the allocation sequence (eg, central telephone; sequentially numbered, opaque, sealed envelopes), describing any steps to conceal the sequence until interventions are assigned | NA (observational study) |  |
| Implementation | 16c | Who will generate the allocation sequence, who will enrol participants, and who will assign participants to interventions | NA (observational study) |  |
| Blinding (masking) | 17a | Who will be blinded after assignment to interventions (eg, trial participants, care providers, outcome assessors, data analysts), and how | NA (observational study) |  |
|  | 17b | If blinded, circumstances under which unblinding is permissible, and procedure for revealing a participant’s allocated intervention during the trial | NA (observational study) |  |
| **Methods: Data collection, management, and analysis** | | | In line with SPIRIT guidelines |  |
| Data collection methods | 18a | Plans for assessment and collection of outcome, baseline, and other trial data, including any related processes to promote data quality (eg, duplicate measurements, training of assessors) and a description of study instruments (eg, questionnaires, laboratory tests) along with their reliability and validity, if known. Reference to where data collection forms can be found, if not in the protocol |  | 10-11 |
|  | 18b | Plans to promote participant retention and complete follow-up, including list of any outcome data to be collected for participants who discontinue or deviate from intervention protocols | We have now added “Participants retention” section under “Data management” section: ” Participant retention: All individuals participating in the study will be provided with a comprehensive report of the clinical assessments performed, which include physical and neurological examinations, MRI results, PSG clinical report, LP clinical results (cell count, protein and glucose levels), biomic blood tests, and cognitive assessments. In accordance with the participants' preferences, the Principal Investigator (PI) will communicate all significant clinical data to their family physician, thereby promoting preventive medicine and the overall health of the participants. Additionally, annual webinars will be arranged to update the participants about the progress of the study and advancements in the related research field. Contact information for both the PI and the study coordinator will be made available to the participants, who will be encouraged to reach out to the study personnel with any questions or concerns.” | 11 |
| Data management | 19 | Plans for data entry, coding, security, and storage, including any related processes to promote data quality (eg, double data entry; range checks for data values). Reference to where details of data management procedures can be found, if not in the protocol |  | 10-11 |
| Statistical methods | 20a | Statistical methods for analysing primary and secondary outcomes. Reference to where other details of the statistical analysis plan can be found, if not in the protocol |  | 9-10 |
|  | 20b | Methods for any additional analyses (eg, subgroup and adjusted analyses) |  | 9-10 |
|  | 20c | Definition of analysis population relating to protocol non-adherence (eg, as randomised analysis), and any statistical methods to handle missing data (eg, multiple imputation) |  | 9-10 |
| **Methods: Monitoring** | | |  |  |
| Data monitoring | 21a | Composition of data monitoring committee (DMC); summary of its role and reporting structure; statement of whether it is independent from the sponsor and competing interests; and reference to where further details about its charter can be found, if not in the protocol. Alternatively, an explanation of why a DMC is not needed | We have added a “Data monitoring” section under “Data management” section stating that: *“The study meets all of the ethical and regulatory requirements for data collection and use and does not require additional oversight.”* | 11 |
|  | 21b | Description of any interim analyses and stopping guidelines, including who will have access to these interim results and make the final decision to terminate the trial | NA (observational study) |  |
| Harms | 22 | Plans for collecting, assessing, reporting, and managing solicited and spontaneously reported adverse events and other unintended effects of trial interventions or trial conduct | NA (observational study) |  |
| Auditing | 23 | Frequency and procedures for auditing trial conduct if any, and whether the process will be independent from investigators and the sponsor | We have now added this to the “Data monitoring” section under “Data management” section: *“The local and the Ministry Of Health (MOH) IRB’s are responsible for conducting periodic audits on the conduct of the trial.”* | 11 |
| Ethics and dissemination | | | In line with SPIRIT guidelines |  |
| Research ethics approval | 24 | Plans for seeking research ethics committee/institutional review board (REC/IRB) approval | We have corrected the wording under the “Ethics approval and consent’ section: “The study was approved by the local ethical committee and is being performed according to the Principles of the Declaration of Helsinki. All participants give their informed written consent prior to participation and after making sure they, or their legal guardian understand the consent and to the PI judgement (N.B.) are eligible to sign an informed consent.  All methods are carried out in accordance with relevant guidelines and regulations. All experimental protocols were approved by the Tel Aviv Medical Center IRB and by the Israeli Ministry of Health IRB (IRB number 0215-18 TLV, protocol version #3, 27-01-19).” | 23 |
| Protocol amendments | 25 | Plans for communicating important protocol modifications (eg, changes to eligibility criteria, outcomes, analyses) to relevant parties (eg, investigators, REC/IRBs, trial participants, trial registries, journals, regulators) | NA |  |
| Consent or assent | 26a | Who will obtain informed consent or assent from potential trial participants or authorised surrogates, and how (see Item 32) | We have now added the following to the Study design section: “*A senior Neurologist (PI or SPI) will obtain the informed consent.”* | 7 |
|  | 26b | Additional consent provisions for collection and use of participant data and biological specimens in ancillary studies, if applicable | NA |  |
| Confidentiality | 27 | How personal information about potential and enrolled participants will be collected, shared, and maintained in order to protect confidentiality before, during, and after the trial |  | 11 |
| Declaration of interests | 28 | Financial and other competing interests for principal investigators for the overall trial and each study site |  | 23 |
| Access to data | 29 | Statement of who will have access to the final trial dataset, and disclosure of contractual agreements that limit such access for investigators |  | 11 |
| Ancillary and post-trial care | 30 | Provisions, if any, for ancillary and post-trial care, and for compensation to those who suffer harm from trial participation | NA |  |
| Dissemination policy | 31a | Plans for investigators and sponsor to communicate trial results to participants, healthcare professionals, the public, and other relevant groups (eg, via publication, reporting in results databases, or other data sharing arrangements), including any publication restrictions | We have now added this under the “Data sharing” section in the “Data management” section: *Communication of trial results will be done via publication in scientific journals and professional conventions, or other data sharing arrangements.* | 11 |
|  | 31b | Authorship eligibility guidelines and any intended use of professional writers |  | 23 |
|  | 31c | Plans, if any, for granting public access to the full protocol, participant-level dataset, and statistical code | NA |  |
| Appendices |  |  |  |  |
| Informed consent materials | 32 | Model consent form and other related documentation given to participants and authorised surrogates | NA |  |
| Biological specimens | 33 | Plans for collection, laboratory evaluation, and storage of biological specimens for genetic or molecular analysis in the current trial and for future use in ancillary studies, if applicable |  | 14-16 |
